# Psychological Capital Moderates the Association Between Social Participation and Depressive Symptoms in Taiwanese University Students

**DOI:** 10.1002/kjm2.70072

**Published:** 2025-06-20

**Authors:** Yi‐Lung Chen, Agata Chudzicka‐Czupała, Cheng‐Fang Yen

**Affiliations:** ^1^ Department of Healthcare Administration Asia University Taichung Taiwan; ^2^ Interdisciplinary Center for Social Activity and Well‐Being Research Faculty of Psychology, SWPS University Katowice Poland; ^3^ Department of Psychiatry Kaohsiung Medical University Hospital Kaohsiung Taiwan; ^4^ Department of Psychiatry School of Medicine, College of Medicine, Kaohsiung Medical University Kaohsiung Taiwan

Dear Editor,

1

Social participation (SP) refers to individual behavior that helps others, such as providing financial support to those in need [1]. SP has been found to correlate with psychological well‐being [2]. Encouraging SP in university students is thought to promote positive psychosocial outcomes. Further study is needed to examine the factors moderating the association between SP and mental health. This study examined the association between SP and depressive symptoms among Taiwanese university students with various levels of psychological capital, which refers to an individual's positive psychological state of development that is characterized by self‐efficacy, optimism, hope, and resilience [2].

A total of 1000 university students responded to an online advertisement posted on the social media platform Dcard from January 2025 to February 2025 and completed the online questionnaire. SP was measured using the Civic Activity Questionnaire (CAQ) [1]. Depressive symptoms were measured using the Center for Epidemiologic Studies Depression Scale (CES‐D) [3]. Psychological capital was measured using the Psychological Capital Questionnaire (PCQ‐12) [2]. A higher total score of the CAQ, CES‐D, and PCQ‐12 indicated greater SP, depressive symptoms, and psychological capital, respectively. Participants' sex, age, financial status, and chronic physical disease status were assessed. The association of SP with depressive symptoms and the moderating effect of psychological capital were examined using multiple linear regression models. Significance was indicated by a *p* value of < 0.05. We used the Johnson–Neyman technique [4] to determine the presence of specific levels of psychological capital at which the conditional effects of SP on depressive symptoms reached statistical significance.

Table 1 presents participants' characteristics and the results of linear regression analysis. The mean (SD) CES‐D score was 14.98 (9.96) (range: 0–56). The results of Model I, II, and III indicate that SP, psychological capital, and the interaction term of SP and psychological capital are negatively and significantly correlated with depressive symptoms. Furthermore, when the Johnson–Neyman technique was applied, a positive association between SP and depressive symptoms was observed for 322 participants with a total PCQ‐12 score < 50. However, no significant association between SP and depressive symptoms was observed for 600 participants with a total PCQ‐12 score ranging between 51 and 65, and a negative association between SP and depressive symptoms was observed for 78 participants with a total PCQ‐12 score > 65.

**TABLE 1:** Associations of social participation with depressive symptoms in multiple linear regression (*N* = 1000).

Variable	*n* (%)	Mean (SD)	Range	Depressive symptoms					
				Model I		Model II		Model III	
				B (SE)	*p*	B (SE)	*p*	B (SE)	*p*
Sex: Male^a^	433 (43.3)			−0.812 (0.578)	0.160	−1.053 (0.482)	0.029	−1.052 (0.479)	0.028
Age		20.77 (1.68)	18–25	0.470 (0.171)	0.006	0.317 (0.143)	0.027	0.332 (0.142)	0.020
Financial situation		1.94 (1.11)	1–4	−3.226 (0.488)	< 0.001	−1.458 (0.416)	< 0.001	−1.435 (0.413)	0.001
Having any chronic physical disease	41 (4.1)			7.138 (1.444)	< 0.001	3.987 (1.213)	0.001	4.122 (1.207)	0.001
Social participation		19.74 (7.93)	8–40	−0.152 (0.036)	< 0.001	0.071 (0.032)	0.026	0.683 (0.174)	< 0.001
Psychological capital		53.04 (9.47)	14–72			−0.575 (0.027)	< 0.001	−0.356 (0.067)	< 0.001
Social participation × Psychological capital								−0.011 (0.003)	< 0.001

^a^ Female as the reference.

According to self‐determination theory [5], individuals have basic psychological needs for autonomy, competence, and relatedness, and that individuals can only flourish when these needs are satisfied [5]. Engagement in SP that fulfills the needs for autonomy and relatedness can enhance subjective well‐being [2]. The moderating effect of psychological capital indicates the complexity of the association between SP and depression. It is possible that having high psychological capital may help university students feel a sense of autonomy, competence, and relatedness from engaging in SP and reduce depression. However, further longitudinal studies are needed to examine the hypothesis.

Participant recruitment was conducted through online advertisements; therefore, our participants may not fully represent the general university student populations in Taiwan. The cross‐sectional design precludes any definitive conclusions regarding the temporal relationships of SP with depression.

## Ethics Statement

This study was approved by the institutional review board of Kaohsiung Medical University Hospital (approval number: KMUHIRB‐E(I)‐20240394).

## Conflicts of Interest

The authors declare no conflicts of interest.

## Data Availability

The data that support the findings of this study are available on request from the corresponding author. The data are not publicly available due to privacy or ethical restrictions.
